# Dengue Virus Enhances Thrombomodulin and ICAM-1 Expression through the Macrophage Migration Inhibitory Factor Induction of the MAPK and PI3K Signaling Pathways

**DOI:** 10.1371/journal.pone.0055018

**Published:** 2013-01-28

**Authors:** Trai-Ming Yeh, Shu-Hsiang Liu, Kao-Chang Lin, Chieh Kuo, Shu-Yun Kuo, Tzuu-Yuan Huang, Yong-Ren Yen, Rong-Kun Wen, Lien-Cheng Chen, Tsai-Feng Fu

**Affiliations:** 1 Department of Medical Technology, National Cheng Kung University, Taiwan, Republic of China; 2 Department of Medical Research, Mackay Memorial Hospital, Taipei, Taiwan, Republic of China; 3 Center of General Education, National Taipei University of Nursing and Health Sciences, Taipei, Taiwan, Republic of China; 4 Department of Neurology, Chi-Mei Medical Center, Tainan, Taiwan, Republic of China; 5 Department of Biotechnology, Southern Taiwan University, Tainan, Taiwan, Republic of China, C; 6 Department of Cardiology, Sin Lau Christian Hospital, Tainan, Taiwan, Republic of China; 7 Graduate Institute of Biomedicine and Biomedical Technology, National Chi Nan University, Nantou, Taiwan, Republic of China; 8 Department of Neurosurgery, Sin Lau Christian Hospital, Tainan, Taiwan, Republic of China; 9 Institute of Medicine, Chung Shan Medical University, Taichung, Taiwan, Republic of China; 10 Taichung Branch, Bureau of Standards, Metrology and Inspection (BSMI), M.O.E.A., Republic of China; 11 Department of Applied Chemistry, National Chi Nan University, Nantou, Taiwan, Republic of China; 12 Department of Medical Technology and Graduate Institute of Biological Science and Technology, Chung Hwa University of Medical Technology, Tainan, Taiwan, Republic of China; 13 Medical Education and Research Center, Sin Lau Christian Hospital, Tainan, Taiwan, Republic of China; 14 School of Medical Laboratory Science and Biotechnology, Taipei Medical University, Taipei, Taiwan, Republic of China; 15 School of Medical Laboratory and Biotechnology, Chung Shan Medical University, Taichung, Taiwan, Republic of China; University Medical Center Freiburg, Germany

## Abstract

Dengue virus (DV) infections cause mild dengue fever (DF) or severe life-threatening dengue hemorrhagic fever (DHF). The mechanisms that cause hemorrhage in DV infections remain poorly understood. Thrombomodulin (TM) is a glycoprotein expressed on the surface of vascular endothelial cells that plays an important role in the thrombin-mediated activation of protein C. Prior studies have shown that the serum levels of soluble TM (sTM) and macrophage migration inhibitory factor (MIF) are significantly increased in DHF patients compared to levels in DF patients or normal controls. In this study, we investigated how MIF and sTM concentrations are enhanced in the plasma of DHF patients and the potential effect of MIF on coagulation through its influence on two factors: thrombomodulin (TM) and intercellular adhesion molecule-1 (ICAM-1) in endothelial cells and monocytes. Recombinant human macrophage migration inhibitory factor (rMIF) was used to treat monocytic THP-1 cells and endothelial HMEC-1 cells or primary HUVEC cells. The subsequent expression of TM and ICAM-1 was assessed by immunofluorescent staining and flow cytometry analysis. Additionally, the co-incubation of THP-1 cells with various cell signaling pathway inhibitors was used to determine the pathways through which MIF mediated its effect. The data provided evidence that severe DV infections induce MIF expression, which in turn stimulates monocytes or endothelial cells to express TM and ICAM-1 via the Erk, JNK MAPK and the PI3K signaling pathways, supporting the idea that MIF may play an important role as a regulator of coagulation.

## Introduction

Dengue virus (DV) infections cause mild dengue fever (DF) or severe life-threatening dengue hemorrhagic fever/dengue shock syndrome (DHF/DSS) [1]. DV infection may be associated with coagulation disorders, resulting in vascular leakage, hemorrhagic diathesis and complement activation [2], [3]. Several studies have suggested that thrombocytopenia and imbalance in the regulation of coagulation and fibrinolysis contribute to the potential for hemorrhage in DHF/DSS [3], [4], [5], [6], [7]. At the same time, due to the complexity of hemostasis, the mechanisms involved in DV-induced hemorrhaging are still not clearly understood.

Thrombomodulin (TM) functions in the anticoagulation pathway by competing with fibrinogen to bind to the thrombin exosite (extended substrate-binding site), inhibiting the generation of fibrin and thereby interfering with coagulation. The thrombin-thrombomodulin complex induces activated protein C (APC). APC, in turn, activates a serine protease that digests the active clotting factors Va and VIIIa, blocking the coagulation pathway and decreasing the formation of thrombin [8], [9]. Along with the previously described clinical characteristics, an increase in the serum level of TM is correlated with disease severity in dengue patients [6], [7], [10], [11]. The underlying mechanisms responsible for anticoagulation in acute dengue infections remain poorly understood. It is known that endothelial cells are susceptible to DV infection both *in vitro* and *in vivo* [12], [13], [14], [15]. DV infection of endothelial cells can enhance platelet adherence and induce tissue plasminogen activator expression, which may contribute to hemorrhaging in DHF/DSS [16], [17]. However, the effect of DV on the expression of molecules involved in the protein C pathway, such as TM, protein S (PS) and endothelial cell protein C receptor (EPCR), in endothelial cells remains unclear.

MIF is a pleiotropic cytokine that plays an important role in modulating inflammatory and immune responses [18], [19]. The MIF protein binds to CD74, a monocytic receptor (the invariant chain of MHC class II) [20], and activates the ERK1/ERK2 protein kinase pathway via CD44 [10]. In some cases, when an organism is infected MIF secretion in the circulation is increased to enhance the activity of a variety of cytokines, including tumor necrosis factor (TNF)-α, interleukin (IL)-1β, IL-2, IL-6, IL-8, IL-12, interferon (IFN)-γ, nitric oxide and COX2 [19], [21], [22], [23], [24]. As shown in our previous clinical study, serum levels of MIF were higher in DHF patients than in DF patients [10]. Recent research has also indicated that in addition to promoting the proliferation of inflammatory cells, recombinant human MIF (rMIF) can stimulate the expression of adhesion molecules, such as ICAM-1 and VCAM-1 in endothelial cells and monocytes, resulting in increased inflammation due to the adhesion of hemocytes [25], [26].

Previous studies have indicated that MIF induces TM and ICAM-1 expression in HMEC-1 cells [10], [26]. Increases in the level of MIF in the serum of dengue virus-infected patients were also observed [6], [7], [11]. Moreover, patients with rheumatoid arthritis (RA) also exhibit increased MIF, and this increase is correlated with TM and MIF production [27]. Based on these observations, we concluded that MIF functions via the CD44 signaling pathway and that anti-CD44 antibodies induce THP-1 cells to transform into monocytes [28], [29]. This conclusion has been supported by a number of studies showing a highly positive correlation between MIF and TM [10], [26], [27]. In spite of the indirect evidence showing that THP-1 cells possess CD44, it remains unknown whether MIF affects signal transduction in monocytes via CD44.

Accordingly, we tried to address this question by exploring the effect of DV on the expression of these molecules in human endothelial cell lines (HMEC-1), primary cells (HUVEC), and monocytes (THP-1) *in vitro*. The results indicated that DV could increase MIF expression to enhance TM and ICAM-1 in monocytes and endothelial cells, which may contribute to the anti-coagulant state of endothelial cells during DV infection. This study sought to further understand the influence of high levels of MIF on TM and ICAM-1 expression in severe DF infections and to determine the pathways that may be involved.

## Results

### MIF and sTM are Increased but PC are Decreased in the Serum of DHF Patients

**Figure 1:**
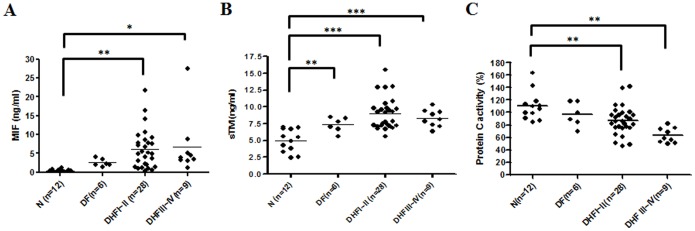
MIF, sTM and PC concentrations are increased in plasma of patients with DHF. Levels of (A) MIF, (B) soluble thrombomodulin (sTM) and (C) Protein C (PC) in patients infected with dengue virus and in control groups. Sera from healthy controls (N, n = 12), dengue fever (DF) patients (n = 12) and dengue hemorrhagic fever patients (DHF) sorted into grades I–II (n = 28) and grades III–IV (n = 9) were assayed for MIF, sTM and PC. The amounts of MIF, sTM and PC in dengue patients were measured using ELISA kits as described in the Materials and Methods. *p<0.1, **p<0.01 and ***p<0.001 compared with corresponding values from normal controls (N). Levels of MIF and sTM were higher than in normal controls, but PC was lower than in the control.

As part of this study, we compared serum levels of MIF and sTM in DV patients with different severities of dengue infection. As described in previous research reports, MIF and sTM levels were increased in the plasma of the DHF patients [7], [10], [30]. According to the criteria of the World Health Organization (WHO), four grades of DHF (or DSS) are recognized, ranging from a least severe grade of I to a most severe grade of IV [31]. Our results demonstrated that serum levels of MIF, as well as of sTM, were significantly increased but PC was decreased in the serum of DV patients, and the levels correlated with disease severity (Fig. 1).

### MIF Expression in DV2-infected Monocytic THP-1 Cells and Primary Endothelial Cells (HUVECs)

**Figure 2:**
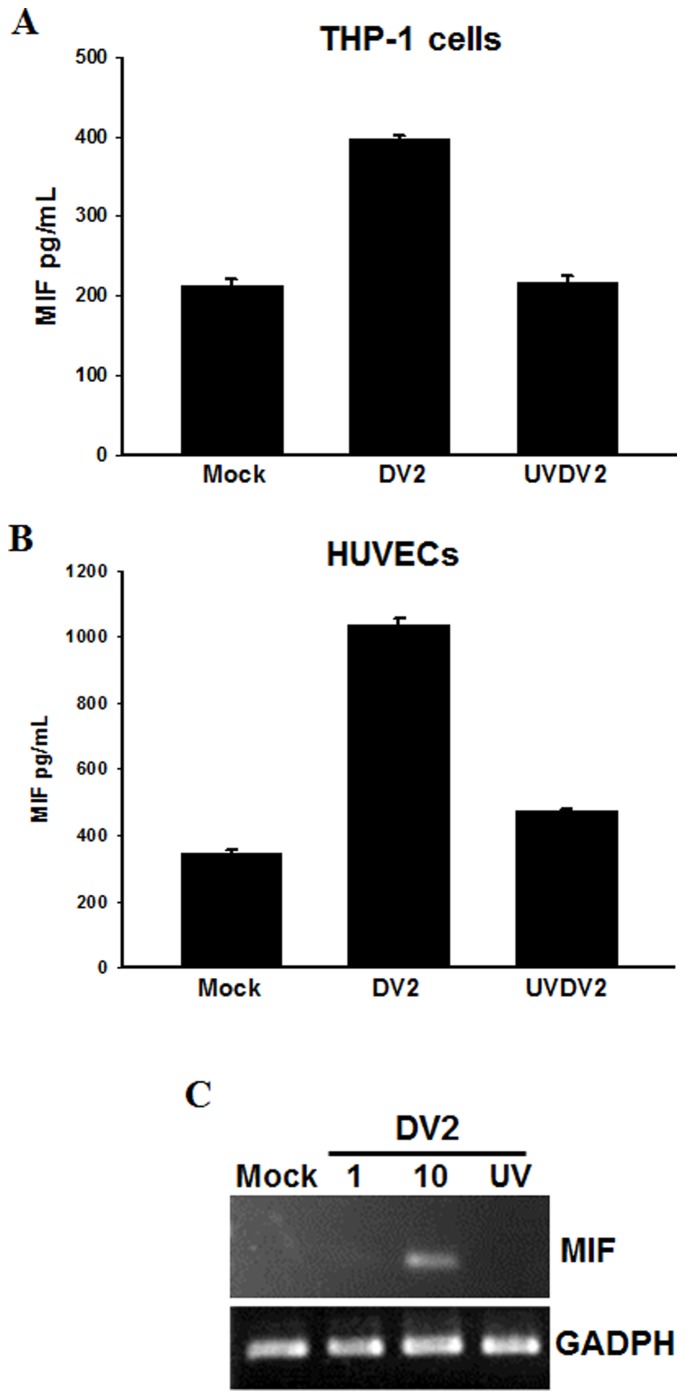
Dengue virus induces MIF production in monocytes and human umbilical cord vein endothelial cells (HUVECs). THP-1 cells or primary HUVECs were infected with DV2 (MOI = 10). (A, B) The concentrations of MIF in culture media were assayed at 48 h post-infection using ELISA kits as described in the Materials and Methods. Controls were uninfected cells (Mock) and cells treated with UV-inactivated DV (UVDV2). (C) DV2 induced MIF mRNA expression in HUVECs. HUVECs were infected with DV2 (MOI = 1 or 10) and incubated for 6 hours. RNA was extracted and the expression of MIF was analyzed by semi-quantitative RT-PCR with specific primers for MIF. The gene expression of GADPH was used as the internal control. Controls were cells without virus (Mock) and cells treated with UV-inactivated DV (UV).

We also explored the effects of DV infection on the production of MIF in human blood mononuclear cells (THP-1) and human umbilical cord vein endothelial cells (HUVECs). To assess whether DV2 infection induces MIF production, we incubated THP-1 cells with DV2 for 48 h at an MOI of 10 and incubated HUVECs with DV2 for 48 h at MOIs of 1 or 10. The ELISA results and analyses showed that DV2 infection will induce MIF production in THP-1 cells and HUVECs but that cells treated with UV-inactivated DV2 (UVDV2) and cells not infected with DV2 (Mock) will not show increased production (Fig. 2A and B). To further confirm the results of the ELISA analysis, we conducted an RT-PCR to examine whether the effect of DV2-infection-induced MIF production is dose-dependent in HUVECs (Fig. 2C). No significant increase in MIF production was detected in mock-infected cells, cells treated with UV-inactivated DV2 (MOI = 10) or cells treated with low dosage DV2 (MOI = 1) stimulation.

### Induction of ICAM-1 Expression by rMIF in Endothelial HUVEC, HMEC-1 Cell Lines and THP-1 Monocytes

**Figure 3:**
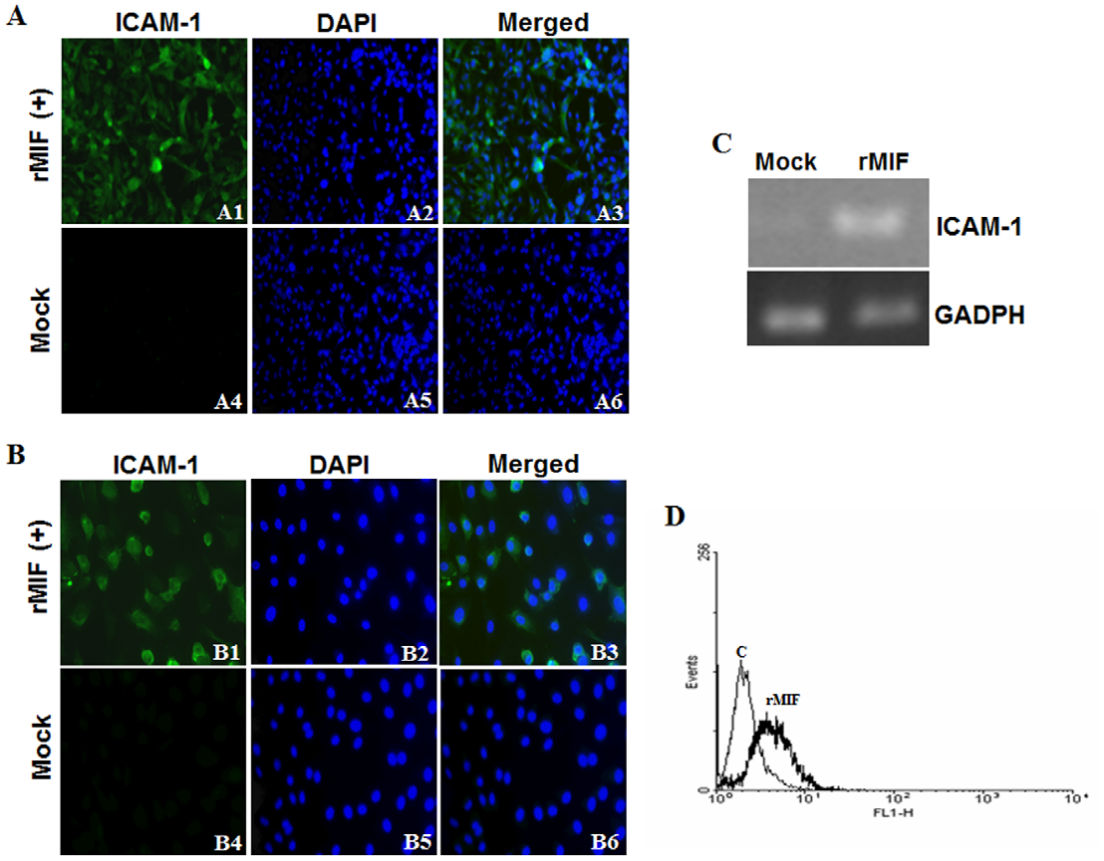
rMIF induces ICAM-1 expression in endothelial cells and monocytes. (A) HMEC-1 (1×10^6^) and (B) HUVEC (1×10^6^) endothelial cells were stimulated with rMIF (0.4 µg/mL) for 24 h. ICAM-1 and nuclei were stained with FITC conjugated anti-ICAM-1 antibody and DAPI, respectively, and observed using fluorescence microscopy at 400× magnification. Negative controls were treated with 0.9% saline (Mock). Thrombin-treated HMEC-1 cells were used as a positive control. THP-1 monocytic cells (2×10^6^) (C and D) were stimulated with rMIF (0.4 µg/mL) for 24 h. (C) RNA was extracted and ICAM-1 expression was analyzed using a semi-quantitative RT-PCR with specific primers for ICAM-1. The gene expression of GADPH was used as the internal control. (D) The expression of ICAM-1 was detected by flow cytometry analysis and compared to the control (without rMIF treatment). Flow cytometry analysis showed increased ICAM-1 expression in THP-1 monocytes following rMIF treatment.

**Figure 4:**
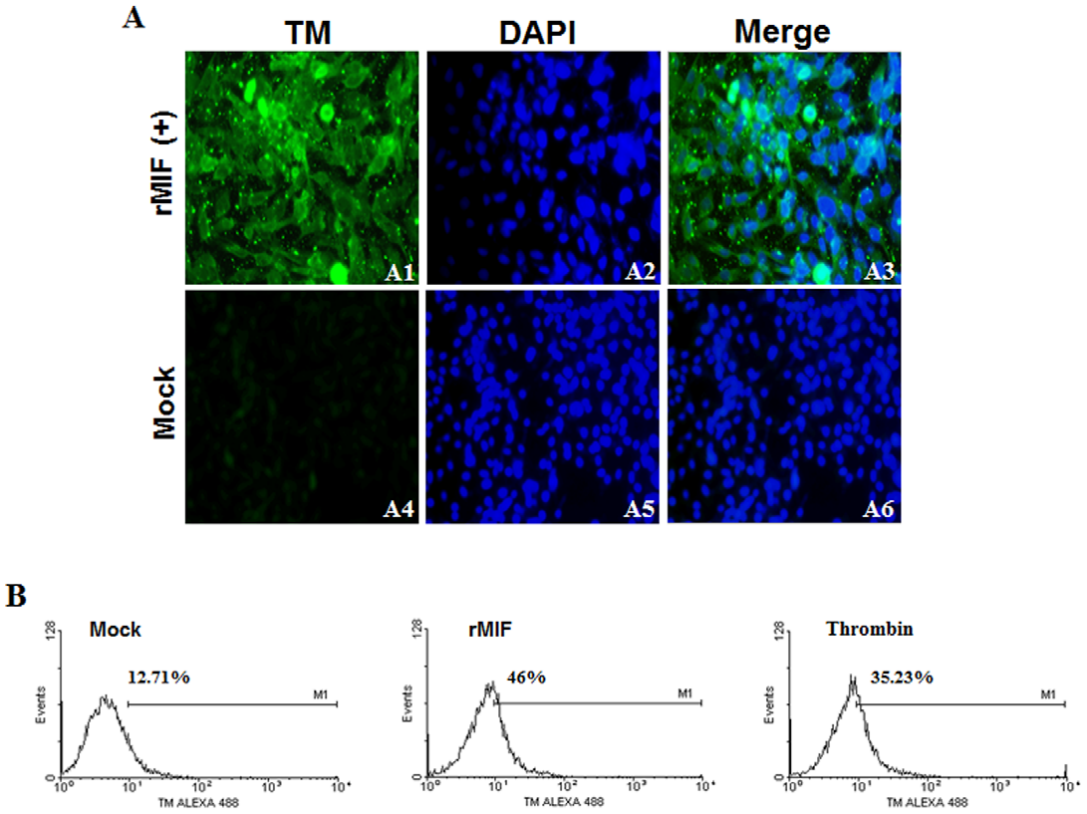
rMIF induces thrombomodulin (TM) expression in endothelial cells. (A) HMEC-1 (1×10^6^) cells were stimulated with rMIF (0.4 µg/mL) for 24 h. TM and nuclei were stained with FITC conjugated anti-TM antibody and DAPI, respectively, and observed using fluorescence microscopy at 400× magnification. The controls were treated with 0.9% saline (Mock). (B) The expression of TM in HMEC-1 cells with/without rMIF treatment was determined using flow cytometry analysis. Cells treated with rMIF showed increased TM expression compared to the control (without). The thrombin-treated cells were used as a positive control.

Serum levels of MIF and sTM were significantly increased in DHF patients [7], [10], [30], and previous studies have shown that MIF can up-regulate ICAM-1 expression in endothelial cell lines (HMEC-1 and HUVECs) [25], [26]. To further characterize the effects of rMIF on the regulation of coagulation, rMIF (0.4 µg/mL) was added to endothelial HMEC-1, HUVEC cells and monocytic THP-1 cells for 24 h to determine its influence on ICAM-1 expression. The immunofluorescence staining results showed strong induction of ICAM-1 expression in HMEC-1 (Fig. 3A, panels 1, 2, 3) and HUVECs (Fig. 3B, panels 1, 2, 3) that were treated with rMIF, and this strong expression of ICAM-1 was not seen in the control cells, which were not treated with rMIF (Mock) (Fig. 4A and B, panels 4, 5, 6). Furthermore, we conducted an RT-PCR to examine the effect of rMIF addition on ICAM-1 expression in THP-1 monocytes. Evidence of increased ICAM-1 expression was observed when rMIF was added to the THP-1 cells, compared to the baseline level of ICAM-1 expression in the controls (Mock) (Fig. 3C). Flow cytometry analysis also showed increased ICAM-1 expression in THP-1 monocytes following rMIF treatment, compared to the control (without rMIF treatment) (Fig. 3D).

### Induction of TM Expression by rMIF in Endothelial HMEC-1 Cell Lines and THP-1 Monocytes

**Figure 5:**
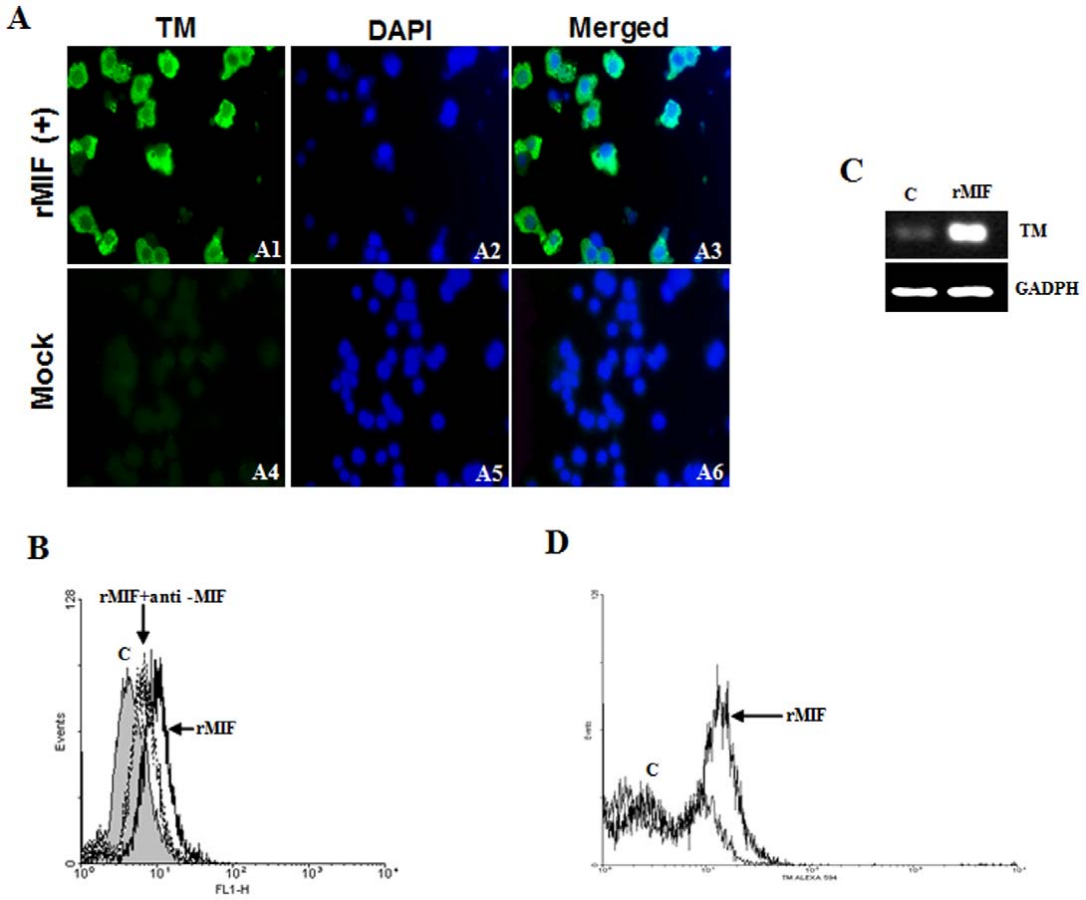
rMIF induces thrombomodulin (TM) expression in monocytes. (A) THP-1 cells (2×10^6^) were stimulated with rMIF (0.4 µg/mL) for 24 h. TM and nuclei were stained with FITC conjugated anti-TM antibody and DAPI, respectively, and observed using fluorescence microscopy at 400× magnification. The controls were treated with 0.9% saline (Mock). (B) Flow cytometry analysis of TM expression in THP-1 cells with/without rMIF treatment. A neutralizing anti-MIF mAb (20 µg/mL) was used to abrogate the MIF-induced TM expression. (C) RNA was extracted and TM expression was analyzed through a semi-quantitative RT-PCR with specific primers for TM. The GADPH was used as the internal control. TM expression was enhanced with rMIF treatment in THP-1 cells, compared to the control (c; without rMIF treatment). (D) Flow cytometry analysis also showed increased TM expression in PBMCs following rMIF treatment.

The influence of rMIF on TM expression was examined by the incubation of rMIF (0.4 µg/mL) with HMEC-1 (Fig. 4) and THP-1 (Fig. 5) cells for 24 h. The immunofluorescence staining results showed a strong induction of TM expression in HMEC-1 cells (Fig. 4A, panels 1, 2, 3) and THP-1 cells (Fig. 5A, panels 1, 2, 3) that were exposed to rMIF, and this strong expression of TM was not seen in the control cells that were not exposed to rMIF (Fig. 4A, panels 4, 5, 6, and Fig. 5A, panels 4, 5, 6). Furthermore, flow cytometry analysis showed that expression of TM was promoted by rMIF in HMEC-1 (Fig. 4B) and THP-1 (Fig. 5B) cells for 24 h. Evidence of increased TM expression was seen when rMIF was added to the THP-1 cells, compared to the baseline levels for cells not treated with rMIF (Mock) and the thrombin-treated HMEC-1 cells used as a positive control (Fig. 5). Similarly, flow cytometry analysis showed that expression of TM was promoted by rMIF in THP-1 cells. The expression of TM was not seen in the control cells or in the THP-1 cells incubated with anti-MIF antibodies, indicating that the influence of rMIF was neutralized (Fig. 5B). To further confirm the results of the flow cytometry analysis, we conducted RT-PCR to examine the effect of rMIF addition on TM expression. Evidence of increased TM expression was seen when rMIF was added to the THP-1 monocytes, compared to the baseline level of TM expression in the controls (Fig. 5C). Flow cytometry analysis also showed increased TM expression in PBMCs following rMIF treatment, compared to the control (Fig. 5D).

### Induction of TM and ICAM-1 Expression by rMIF in THP-1 Cells through the Erk, JNK MAPK and PI3K Pathways

**Figure 6:**
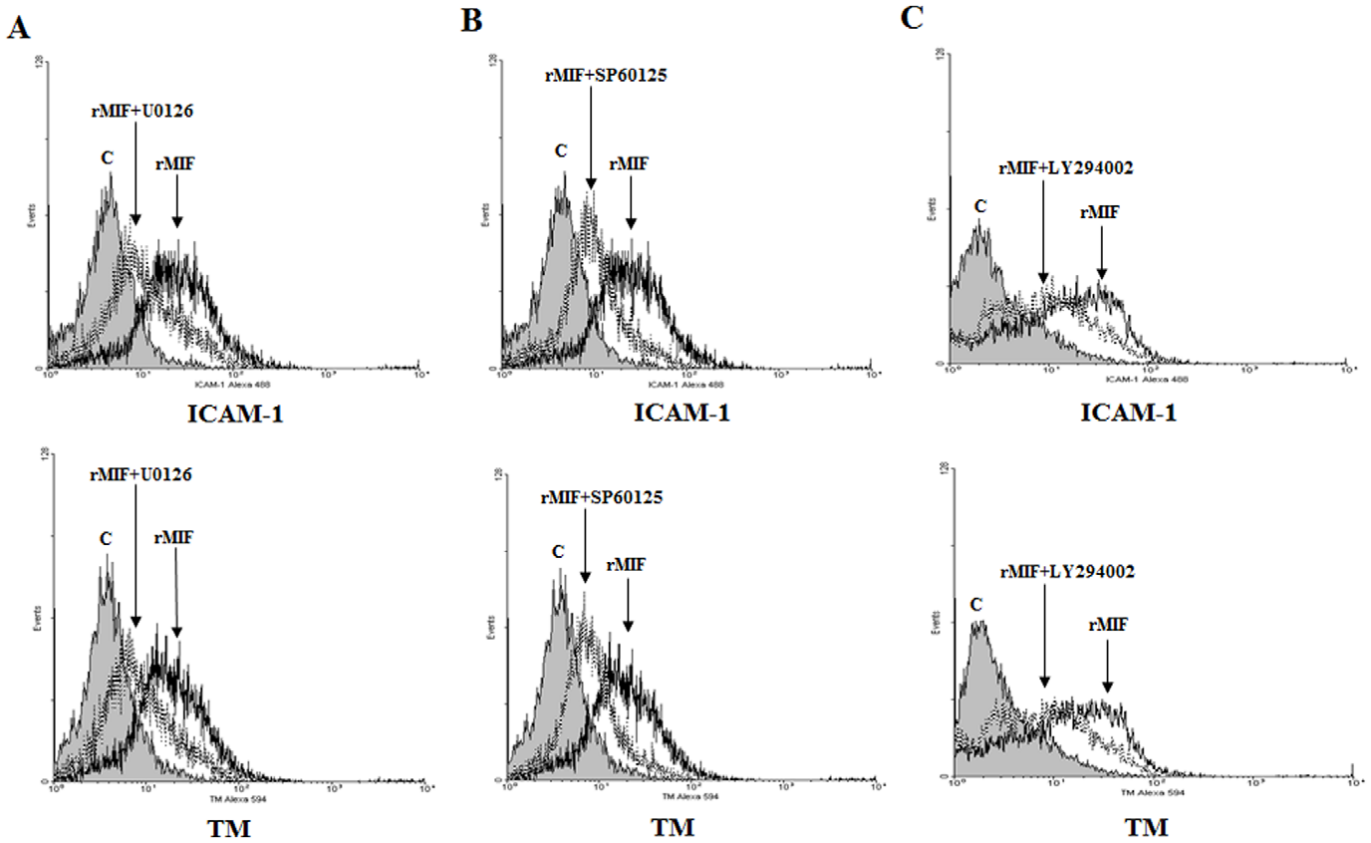
rMIF enhances ICAM-1 and TM expression via the Erk, JNK MAPK and the PI3K signaling pathways in THP-1 cells. (A) Erk inhibitor U0126, (B) JNK inhibitor SP60125, or (C) PI3K inhibitor LY294002 at 20 µM in DMSO was added to the THP-1 cell culture 30 min before and throughout rMIF treatment. The cells (2×10^6^) were stimulated with rMIF (0.4 µg/mL) for 24 h. The expression of ICAM-1 (Left panel) and TM (Right panel) was assessed by flow cytometry analysis. The rMIF-treated cells were compared to the controls.

To understand the signaling pathway used to control TM and ICAM-1 expression by rMIF, U0126 (an Erk-1, -2 kinase inhibitor) was utilized to treat THP-1 cells prior to the addition of rMIF. The resultant expression of ICAM-1 and TM was inhibited, suggesting that rMIF could enhance the expression of TM and ICAM-1 through the Erk-1, Erk-2 pathway (Fig. 6A). Similar results were obtained when U0126 was replaced by SP600125 (a JNK kinase inhibitor) (Fig. 6B) or LY294002 (a PI-3 kinase inhibitor) (Fig. 6C), demonstrating that rMIF enhances expression of TM through the JNK and PI-3 signaling pathways.

## Discussion

MIF can directly or indirectly promote the expression of many pro-inflammatory molecules, including TNF-α, IFN-γ, IL-1β, IL-2, IL-6, IL-8, nitric oxide and COX2 [21], [22]. Previous studies have shown that the anticoagulant factors related to the APC pathway in the serum of a patient infected with Dengue virus, including soluble thrombomodulin (sTM), would increase with the seriousness of any resulting DHF, while the levels of PC and PS would decrease [6], [7], [10], [11]. It was inferred that this increase of TM in serum possibly resulted from an increase of biosyntheses or damage to endothelial cells, causing TM to peel off the cell membrane, drift into the serum and further affect the balance of the inflammatory effect. However, it is still uncertain whether the soluble thrombomodulin (sTM) in plasma has the physiological function of regulating APC anticoagulant. According to the findings of two research teams, the function of sTM in activating protein C was only approximately 30–50% when compared to the sTM in plasma with the membrane-bound form of TM [32], [33]. Furthermore, the contents of PC and PS in the serum were found to decrease markedly as the disease severity of DHF in patients increased. This result may have been observed because the host maintained an abnormal bleeding state and the increasing generation of PC/PS after infection by Dengue virus could not overcome the degradation rate, so the PC/PS levels appeared to be decreased. To demonstrate the positive correlations between the contents of MIF and TM in the serum of DHF patients [10], human genetic recombinant MIF (rMIF) was used to study the effects of rMIF on clotting factors, anticlotting factors and the expression of adhesion molecules.

Previous research has shown that rMIF can induce ICAM-1 and TM expression in endothelial cells [25], [26]. In this study, we further demonstrated that rMIF could induce the expression of ICAM-1 and TM in endothelial cell lines, HUVECs and HMEC-1 cells, and monocytic THP-1 cells. MIF is indeed related to the high performance of ICAM-1 and TM. Taken together, the factors that facilitate TM performance include thrombin [34], vascular endothelial growth factor [35], histamine, dibutyryl cyclic adenosine monophosphate, retinoic acid, theophylline, heat shock protein and statins [8], while the factors that inhibit the performance include fluid shear stress [36], hypoxia, oxidized low-density lipoproteins and TGF-β [37], [38]. Moreover, some evidence has also indicated that the endotoxin LPS, and the cytokines TNF-α and interleukin-1β, have regulatory effects on TM expression in phagocytes [39] but inhibit TM expression in endothelial cells. Recent research has also indicated that LPS affects monocytes by inhibiting TM expression through TNF-α, and this effect appears to be serum-dependent [40].

**Figure 7:**
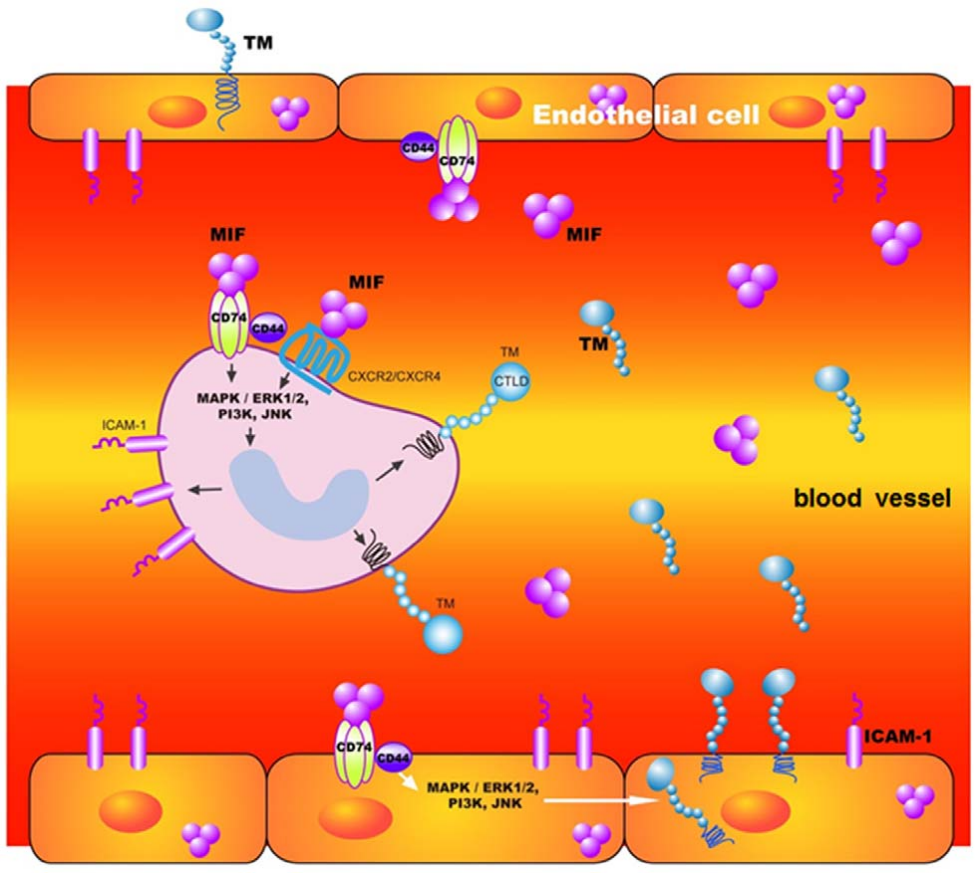
Schematic diagram of the roles of MIF in DV-infected patients’ circulation. DV can stimulate monocytes and endothelial cells to express MIF. MIF signals through binding to a functional receptor complex CD74/CD44 and G-protein-coupled chemokine receptors (CXCR2/CXCR4) individually [20]. Following signaling may activate PI3K/MEK/ERK and downstream JNK signaling pathways, resulting in TM and ICAM-1 expression and causing soluble TM levels to increase in plasma.

We have compiled the information about the roles of MIF in DV-infected patients’ circulation in a schematic diagram (Fig. 7). Both this study and past research indicate that rMIF could induce the expression of ICAM-1 and TM in endothelial cells and monocytes. However, this study investigated the signaling pathway by which rMIF stimulates cells to express TM and ICAM-1. For example, MIF could bind to the CD74 receptor (invariant chain of MHC class II) on cell membranes [20] and activate ERK1/ERK2 through CD44 transferring signals [28]. In this study, ERK1/ERK2, JNK and PI-3K inhibitors were used to treat THP-1 cells, demonstrating that they blocked rMIF stimulation of TM and ICAM-1 expression. Previous research has also indicated that rMIF could induce the production of ICAM-1 through such signal transduction pathways [25]. It could therefore be inferred that rMIF stimulates monocytes to generate TM and ICAM-1 through the ERK1/ERK2 MAPK pathway, which is distinct from statin-regulated TM expression through the inhibition of Rac/Cdc2 (small G proteins of the Rho family) [41], [42]. Some reports have also indicated that the inflammatory cytokine TNF-α inhibits TM expression by affecting the activation of NF-kB [43], [44]. TM has both anti-inflammatory and anticoagulant functions [8], while MIF is an important cytokine for inflammation. Therefore, we suggest and propose a pathogenic mechanism of severe DHF/DSS that explains how DV or MIF induce TM expression in endothelial cells and monocytes. Induction of TM expression may facilitate the formation of the TM-thrombin protein C complex to activated protein (APC) and cause an imbalance between coagulation and anticoagulation and the antithrombotic/anti-inflammatory and procoagulant/proinflammatory states during DHF/DSS. In this study, the effect of MIF on ICAM-1 and TM expression was marked, but the balance in their relationships is worth further exploration and discussion.

## Materials and Methods

### ELISA for MIF, TM and PC Measurement

The levels of MIF were measured using a commercially available ELISA kit (R & D Systems, Minneapolis, MN), as previously described [10]. The TM levels were determined using TM ELISA kit (American diagnostic, Hauppauge, NY) and PC levels were determined using a PC ELISA kit (Helena Laboratories, Beaumont, TX) according to the manufacturer’s instructions. The concentrations of MIF, TM and PC were calculated according to a standard curve. Samples with levels above the maximum optical density were diluted and retested.

### Cell Culture and Other Reagents

Immortal monocytic cells (THP-1 cells; ATCC) and PBMCs were cultured at 37°C in 5% CO_2_ in an RPMI 1640 medium with 10% heat-inactivated fetal calf serum (Gibco BRL). Human microvascular endothelial cells (HMEC-1 cells) were cultured at 37°C in 5% CO_2_ in an MCDB131 medium with 10% heat-inactivated fetal calf serum (Gibco BRL), and human umbilical vein endothelial cells (HUVECs) were purchased from Bioresource Collection and Research Center (BCRC, Taiwan) and were cultured as described previously [15]. All other reagents, including U0126 (Erk inhibitor), SP60125 (JNK inhibitor) and LY294002 (PI3 kinase inhibitor), were purchased from Sigma unless otherwise indicated.

### Preparation of Virus Stock and Virus Titration

Dengue type 2 (strains PL0046 and 16681) was propagated in C6/36 cells (ATCC) [45], [46]. Briefly, monolayers of C6/36 were inoculated with the dengue virus at a multiplicity of infection (MOI) of 0.01 and incubated at 26°C in 5% CO_2_ for 5 days. The culture medium was harvested and cell debris was removed by centrifugation at 900×*g* for 10 min. After further centrifugation at 16,000×*g* for 10 min, the virus supernatant was collected and stored at −70°C until use. Virus titer was determined by plaque assay using the BHK-21 cell line. Briefly, a 10-fold serial dilution of virus was added to BHK-21 monolayer and then incubated at 37°C in 5% CO_2_ for 5 days. Plaque numbers were counted after crystal violet staining.

### DV Infection

Approximately 1×10^5^ cells were seeded into each well of 12-well tissue-culture plates (Falcon, Heleona, MT). After overnight incubation, DV was added to the cells at the suitable MOI and allowed to adsorb for 2 h. Unbound viruses were removed by washing with PBS. Infected cells and culture supernatants were collected at different time intervals after infection. Cells without infection (medium alone, mock) or inoculated with UV-inactivated dengue virus (UVDV) were used as controls.

### Immunofluorescent Staining and Flow Cytometric Assay

Cells (2×10^6^) were treated with rMIF (0.4 µg/ml) or DV infection for a suitable incubation time, fixed with 4% paraformaldehyde for 30 min and permeabilized with 0.5% Triton X-100 for 10 min. The permeabilized cells were washed with phosphate buffered saline (PBS) and blocked with 0.05% bovine serum albumin in PBS. The cells were stained with primary antibody at 4°C for 1 h. After being washed, the cells were incubated with a secondary antibody and observed under a fluorescence microscope (Olympus) or were used for flow cytometric analysis (FACSCalibur, Becton-Dickinson) and analyzed using WinMDI software. Mouse anti-thrombomodulin antibody (Santa Cruz Biotechnology; Santa Cruz, CA), rabbit anti-MIF antibody (Santa Cruz Biotechnology) and mouse anti-ICAM-1 antibody (Santa Cruz Biotechnology) were used as primary antibodies. FITC-conjugated goat-polyclonal anti-mouse IgG antibody (1∶200 dilution; Jackson ImmunoResearch, West Grove, PA), Alexa 488-conjugated goat-polyclonal anti-Rabbit IgG antibody (1∶200 dilution; Invitrogen) and Alexa 594-conjugated goat-polyclonal anti-Rabbit IgG antibody (1∶200 dilution; Invitrogen) were used as secondary antibodies. DAPI (4′, 6′-diamidino-2-phenylindole) was used as a non-specific nuclear (DNA) stain.

### Reverse-transcription Polymerase Chain Reaction (RT-PCR)

RNA was extracted from HUVEC or THP-1 cells using a Trizol-based (Life Technologies Inc., Rockville, MD) isolation method and quantified at 260 nm. Reverse-transcription (RT) was performed using a kit (Invitrogen, Carlsbad, CA) according to the manufacturer’s instructions. Glyceraldehyde-3-phosphate dehydrogenase (GAPDH) was used as a control. A forward primer, 5′-CCCTTCATTGACCTC-3′, and a reverse primer, 5′-GTCATCCATGACAACTTTGG-3′, were employed to amplify for GAPDH cDNA; a forward primer, 5′-CTCGAGCTGCAGGAACCAATACCCAT-3′, and a reverse primer, 5′-AAGCTTGGCATGATGGCAGAAGGACC-3′, were employed to amplify for MIF cDNA; a forward primer, 5′-CCG GAAGGTGTATGAACTG-3′, and a reverse primer, 5′-TCCATGGTGATCTCTCCTC-3′, were employed to amplify for ICAM-1 cDNA; and a forward primer, 5′-GAGGACGTGGATGACTGCAT-3′, and a reverse primer, 5′-TCACAGTCGGTGCCAATGT G-3′, were employed to amplify for TM cDNA. After reverse transcription, the total reaction volume was set at 20 µL, with 4 µL of RT product, 2.5 units of Taq DNA polymerase, 20 µM of dNTP, 0.1 µM of primer and 1 unit of DNA polymerase buffer (Promega, Madison, WI). The reaction mixture was incubated in a thermocycler (Perkin-Elmer, Fremont, CA) programmed to pre-denature at 95°C for 5 min, denature at 95°C for 30 s, anneal at 56°C for 45 s and extend at 72°C for 1 min, for a total of 35 cycles. After the last cycle, the resulting mixture was incubated at 72°C for 7 min and cooled to 4°C. The PCR products were analyzed on ethidium bromide-stained agarose gels.

### Cloning and Expression of Recombinant MIF (rMIF) Protein

Human full-length MIF cDNA (Genbank Data Bank Accession number Z23063), coding amino acids 1–115, was amplified by PCR from a Jurkat T-cell line using Advantage II polymerase (Clontech Laboratories; Mountain View, CA), a forward primer, 5′-CATATGCCGATGTTCATCGTAAACAC-3′, and a reverse primer, 5′- CTCGAGTAGGCGAAGGTGGAGTT-3′. The PCR product was gel-purified, digested with restriction enzymes *Nde*I and *Xho*I and ligated into the *Nde*I/*Xho*I sites of the pET 21a expression vector (EMD Biosciences; Darmstadt, Germany) to produce pET21a-MIF; the nucleotide sequence was confirmed by DNA sequence analysis. Large-scale purification of rMIF was performed as previously described [47]. Briefly, BL21 (DE3) cells (EMD BioSciences) were transformed with pET21a-MIF, grown to A_600 nm_ of 0.7 and induced with isopropyl-1-thio-D-galactopyranoside (IPTG; 5 mM final concentration) at 37°C for 8 h. The cells were then lysed with binding buffer (100 mM NaCl, pH 8.0) and centrifuged. After centrifugation, the fusion protein was purified from a MagExtractor-His-tag (Toyobo; Osaka, Japan) according to the manufacturer’s instructions.
